# Primary Prevention of Canine Atopic Dermatitis: Breaking the Cycle—A Narrative Review

**DOI:** 10.3390/vetsci10110659

**Published:** 2023-11-16

**Authors:** Beatriz Fernandes, Susana Alves, Vanessa Schmidt, Ana Filipa Bizarro, Marta Pinto, Hugo Pereira, Joana Marto, Ana Mafalda Lourenço

**Affiliations:** 1 CIISA—Centre for Interdisciplinary Research in Animal Health, Faculty of Veterinary Medicine, University of Lisbon, 1649-004 Lisbon, Portugal; 2 Associate Laboratory for Animal and Veterinary Sciences (AL4AnimalS), 1300-477 Lisbon, Portugal; 3 Research Institute for Medicine (iMed.ULisboa), Faculty of Pharmacy, Universidade de Lisboa, 1600-277 Lisbon, Portugal; 4 School of Veterinary Science, University of Liverpool, Liverpool L69 3GH, UK

**Keywords:** canine atopic dermatitis, skin barrier dysfunction, skin barrier repair, canine atopic dermatitis primary prevention, preventive medicine

## Abstract

**Simple Summary:**

This paper addresses the pressing issue of canine atopic dermatitis, a common and distressing skin condition in dogs. It affects up to 30% of dogs, causing severe itching and skin problems. Additionally, it leads to secondary infections, further complicating treatment. This condition significantly reduces the quality of life for both dogs and their owners, causing emotional and financial strain. This paper emphasizes the need for prevention strategies for this disease, rather than just treating the symptoms. While treatments exist, they often come with limitations and can be expensive. It also emphasizes the importance of understanding the skin barrier’s role in canine atopic dermatitis development. This paper suggests that focusing on preventing this condition in the first place would be more effective and cost-efficient. Drawing parallels between canine atopic dermatitis and its human counterpart, this paper highlights the potential for shared prevention strategies. The authors propose that restoring the skin barrier before the disease’s mechanisms can lead to a vicious cycle of further damage could be a key approach to prevention. Overall, research regarding the primary prevention of canine atopic dermatitis has the potential to greatly improve the well-being of dogs and their owners by offering effective and accessible preventive measures.

**Abstract:**

Canine atopic dermatitis (cAD) is a common and distressing skin condition in dogs, affecting up to 30% of the canine population. It not only impacts their quality of life but also that of their owners. Like human atopic dermatitis (hAD), cAD has a complex pathogenesis, including genetic and environmental factors. Current treatments focus on managing clinical signs, but they can be costly and have limitations. This article emphasizes the importance of preventing cAD from developing in the first place. Understanding the role of the skin’s protective barrier is crucial, as its dysfunction plays a vital role in both hAD and cAD. hAD prevention studies have shown promising results in enhancing the skin barrier, but more research is needed to support more robust conclusions. While hAD primary prevention is currently a focal point of intensive investigation in human medicine, research on cAD primary prevention remains under-researched and almost non-existent. Pioneering effective prevention strategies for cAD holds immense potential to enhance the quality of life for both dogs and their owners. Additionally, it bears the promise of a translational impact on human research. Hence, further exploration of this crucial topic is not only relevant but also timely and imperative, warranting support and encouragement.

## 1. Introduction

Canine atopic dermatitis (cAD) is one of the most prevalent, distressing, chronic, and allergic skin diseases, affecting approximately 20–30% of dogs [1,2]. The most common and clinically significant feature of cAD is moderate to severe pruritus, often accompanied by dermatological lesions, such as erythema, self-induced alopecia, excoriations, hyperpigmentation, and lichenification [3,4,5]. These lesions can vary in body location, extension, and severity [3,4,5]. Furthermore, the affected dogs’ skin is frequently affected by secondary infections, exacerbating both pruritus and lesion severity, leading to an unpleasant skin odour and complicating clinical management [6,7]. cAD is the most common primary cause of otitis externa [8], a highly prevalent condition in atopic dogs, with reported incidences ranging from 17 to 80% of cAD cases [9,10]. Around 60% of atopic dogs also suffer from concomitant allergic conjunctivitis, with the severity of ocular signs being significantly correlated with eye pruritus and skin lesion scores in the head region [11]. More recently, a study found that pruritus severity was associated with a higher frequency of undesired behaviours [12]. These included mounting, chewing unsuitable objects, hyperactivity/restlessness, coprophagia, begging for and stealing food, attention seeking, excitability, excessive grooming, reduced trainability, and touch sensitivity [12]. These behaviours are undesirable and often considered by behaviour biologists as indicative of stress, suggesting that atopic dogs may be experiencing pruritus-induced low-level chronic psychological stress [12].

cAD’s incurable and relentless nature, associated with its wax and wane behaviour (periods of flares intertwined with periods of improvement), leads to significant quality of life (QoL) loss for both dogs and their owners [2,13,14]. The QoL of these animals exhibits a negative correlation with their pruritus scores, meaning that as pruritus intensifies, their QoL scores diminish [15,16]. Among the aspects of the dogs’ lives that are most affected by cAD are alterations in behaviour or mood, limitations in play or work activities, the added burden of treatment administration [16], and sleep disturbance [15]. In the case of the owners, the areas of their lives that are most impacted include increased financial expenditure, loss of time, and heightened emotional and physical distress [15,16]. A recent study further highlighted that managing cAD can result in caregiver burden [17], a psychological phenomenon characterised by the level of distress experienced while providing care for patients, which encompasses various aspects of caregivers’ physical, emotional, social, financial, and daily life domains” [18]. This phenomenon has also been identified in individuals caring for relatives with dementia [19] or cancer [20].

Also, cAD affects many dogs from an early age, and although all efforts are made to prevent crises [21], these are still common and frustrating for all. The expensive diagnostic process, frequent crises, and lifelong treatment pose a significant challenge for owners, pets, and veterinarians [22]. Additionally, the prevalence of hypersensitivities seems to be increasing [22], which suggests that this frustrating disease may become an even more common problem in the future.

cAD is also very similar to its human counterpart (hAD), particularly regarding its spontaneous development, the type and distribution of skin lesions, the presence of immune dysfunction, skin barrier defects, and frequent complications caused by secondary infections. Additionally, for many centuries, dogs have shared both man’s environment and lifestyle and, consequently, share some exposome risk factors for atopic dermatitis [23,24]. Thus, both cAD and hAD can be used as a model for the other regarding this disease’s management and prevention strategies [23,25,26].

## 2. cAD Clinical Management

cAD clinical management is a new two-phased treatment strategy [27]. In phase I, the “reactive therapy”, an atopic dog with existing skin lesions and itch (flare) is treated with quick-acting and broad-targeting drugs, such as glucocorticoids or oclacitinib, to induce clinical remission [27]. Phase II, the “proactive therapy”, aims to prevent the occurrence of new flares or at least reduce their frequency and severity [27]. More directed and specific tools such as allergen immunotherapy (AIT) and other pharmacological interventions, such as cyclosporin, injectable biologicals (lokivetmab), or topical product application, can be used for this purpose [27].

However, all management choices present various relevant limitations. The systemic anti-inflammatory and antipruritic treatments that are available are only symptomatic; in other words, they “hide” cAD clinical manifestations and are all associated with potentially adverse effects [28]. Moreover, this symptomatic approach usually requires a multimodal strategy [28], frequently demanding the administration of two or more costly medications with varying frequencies and dosages over time [28,29]. Thus, this approach is not only complex for both veterinarians and owners but, unfortunately, it is also often prohibitively expensive. For example, a recent study conducted in the Teaching Hospital’s Dermatology Service of the University of Lisbon’s Faculty of Veterinary Medicine concluded that, despite 92.5% of owners being satisfied with the use of oclacitinib as a treatment tool, 75% presented worry regarding the cost and 42.5% admitted that they had to limit other expenses to pay for it. Despite an alternative protocol that successfully halved the costs for 71% of dogs on oclacitinib, this treatment often remains unaffordable [30].

Allergen immunotherapy is the only treatment that can effectively change the pathogenic mechanisms of cAD and is commonly available in subcutaneous and sublingual protocols [31]. Its limitations include the need to improve efficacy and safety, decrease the time to efficacy onset, and, most importantly, improve patient compliance [31], the most predominant factor in therapy failure [32]. New non-invasive, safe, effective, painless, and easy-to-use, at-home vaccine delivery routes that promote compliance are needed. Epicutaneous immunotherapy (EPIT) is a promising alternative that takes advantage of the skin’s unique immunological features and high accessibility. Recently, a pilot study emphasised EPIT’s great potential as an effective, well-tolerated, and safe cAD treatment [33].

The primary objective of this paper is to concentrate on primary prevention strategies for atopic dermatitis, as opposed to providing an extensive examination of the current understanding and clinical management of cAD. This latter aspect has been comprehensively addressed in recent and engaging papers [7,34,35,36].

## 3. What Is Prevention?

Despite advances in atopic dermatitis (AD) treatments in both species, research prevention has been slow in human research [37] and almost absent in canine research. This article attempts to critically review the current state of science in the prevention of cAD, comparing it with hAD.

Firstly, some key concepts will be defined. In hAD, primary prevention (PP) usually refers to intervening before the disease occurs. Secondary prevention (SP) refers to acting when the disease has been recognised but is not causing suffering yet, whereas tertiary prevention (TP) involves reducing symptoms or improving the quality of life to prevent further deterioration [38].

Comparatively, in cAD, TP corresponds to the treatment of flares, or “reactive therapy”; SP corresponds to the prevention of flares, or “proactive therapy”; and PP corresponds to strategies or interventions that are implemented to avoid the development of the disease altogether. However, while there is extensive research to develop and improve existing treatments [7,27,28,30,33,35,39,40,41,42,43] and even to develop proactive strategies [21,44], cAD primary prevention is entirely neglected. Despite Marsella (2013) suggesting that skin barrier repair interventions could prove beneficial when initiated in early life in cAD’s predisposed breeds [45], research on these strategies has not been pursued. However, it is reasonable to assume that AD prevention is a more logical and cost-effective way to manage its burden than focusing research entirely on its treatment [37]. The logic of this approach is intuitive: it is better to prevent diseases than to concentrate resources on treating them when treatment may be too late to be effective [46]. Such is the case of cAD, for it is an incurable disease, and its treatment only aids already suffering patients after a long chain of irrevocable pathological events. In fact, a cost-effective analysis has concluded that using emollients as a preventive therapy for hAD is attractive from both medical and economic perspectives [47]. Additionally, disease prevention can modulate the disease’s prevalence and severity [46], which is also a desirable goal in conditions with a significant impact on patients’ and caregivers’ QoL.

### 3.1. Knowledge of AD Pathogenesis: Is It Important?

An essential step in preventing any disease is a thorough understanding of the risk factors that can be manipulated. The pathogenesis of cAD is not fully understood, but it is believed to involve complex interactions between genetic and environmental factors that lead to epidermal barrier dysfunction, immune dysregulation, and cutaneous dysbiosis [7]. Recent hAD studies suggest that skin barrier impairment, cutaneous dysbiosis, and immunological dysfunction are indeed the main factors involved in AD pathogenesis [48,49], with the first possibly being the etiological factor that triggers the disease [50,51].

Indeed, in human medicine, there has been a slow shift from the classic AD paradigm, which gives greater weight to immunological dysregulation (inside–outside), to a new paradigm where more relevance is given to the possible primary skin barrier defects, which are then further aggravated by inflammation (outside–inside–outside) [52,53,54]. Defects in the protein and lipid content of the stratum corneum (SC) contribute to the skin barrier’s impairment [49]. These defects allow the skin microbiome and environmental exposome to penetrate the skin barrier, inducing immune dysregulation (imbalance towards a type 2 immune response, with the overexpression of Th2 and Th22 cytokines), which further exacerbate skin barrier defects, worsening its barrier function, leading to a vicious cycle [49]. Regarding veterinary medicine, a few studies have also suggested that this skin barrier dysfunction is a critical aspect of cAD and that further research should focus on developing skin barrier restoration strategies that are specifically tailored to this disease [14,55].

Like in hAD [49], early cutaneous barrier dysfunction in dogs leads to an increased transepidermal water loss (TEWL) [56], promotes cutaneous dysbiosis [14,35,55], and allows the penetration of allergens and pathogens with the consequent initiation of abnormal inflammatory and allergic responses in the skin [14].

The significance of the cutaneous barrier’s dysfunction to the development of AD is heightened if we consider that atopic children’s skin barrier dysfunction is already detectable soon after birth, preceding AD development [57,58,59,60], and that allergen sensitisation can occur via epicutaneous exposure in both species [61,62], even in non-genetically predisposed dogs [63].

The skin barrier is localised on the uppermost layer of the epidermis, the cornified layer, SC, which is composed of keratinocytes and an intercellular lipidic mantle composed of cholesterol, free fatty acids, and ceramides, among others [49,64,65].

Several cAD studies have established abnormalities in the SC lipids [50,51,56,66,67,68,69,70,71,72,73,74]. The deposition of the intercellular lipid mantle is altered, presenting high lipidic disorganisation, discontinuities and a reduced number of lipids relative to the skin of healthy dogs [56,66,67,69]. These abnormalities are present in lesional and nonlesional skin of atopic dogs and are also exacerbated by allergen challenges [69].

Besides the amount of both total lipids and fatty acids being altered, ceramides have also been found to be significantly reduced in comparison to healthy dogs, with lesional skin usually being more affected than nonlesional skin [56,68,71,72,73,74,75]. Regarding ceramides, which represent the largest group of intercellular lipids in the SC [76], canine studies have shown mixed results, from global decreases in all classes of ceramides to reductions only in specific subclasses [50,68,71,73,74,75].

In fact, solid conclusions are yet to be established, since SC lipid research is highly complex [77,78]. These mixed results can have various causes, from individual variations due to age, gender, body sites, disease status, or breeds, as suggested in some studies [70,73,74,79,80,81], to technical differences or limitations. Given that the methods for both the collection and analysis of SC lipids vary significantly between studies, it would not be unreasonable to partially attribute these mixed results to the latter [73,74,77].

Perhaps it can be expected that this limitation will be shortly overcome due to the increasingly expeditious evolution of technology and the development of more sophisticated methods. Furthermore, it would undoubtedly be beneficial to establish a consensus on a defined process of measuring SC lipids, similar to the established outcome measurements in therapeutic clinical trials enrolling atopic dogs [82].

All pathogenic theoretical knowledge should be translated into therapeutic practice [48], and thus, these recent pathogenic findings of the cutaneous barrier present an unmissable opportunity to explore AD primary prevention strategies. Much-needed, desirable, and promising research is already in progress in hAD, with cAD severely lagging behind.

### 3.2. Primary Prevention Strategies in Humans

The number of hAD prevention studies has increased over the last decade [37]. These studies can be divided into three categories: interventions that are ingested by mothers or infants, which include exclusive breastfeeding, the delayed or early introduction of foods other than milk, dietary restrictions, and dietary supplements; interventions directed at the external skin surface, which include attempts to reduce airborne allergens such as house dust mites at the time of birth, increasing exposure to environmental protector factors, and measures to enhance the skin barrier; and, finally, combined approaches [37].

The most investigated strategies are probiotics and interventions to enhance the skin barrier [37]. The ingestion of probiotics, prebiotics, or synbiotics is an hAD prevention strategy with increasingly supporting evidence [37,83]. This strategy has been steadily showing moderate benefits and good safety, leading the World Allergy Organisation guideline panel to suggest using probiotics in women, either pregnant with or breastfeeding infants at high risk of developing allergies, and infants at high risk of developing allergies [37].

The mounting scientific evidence regarding AD pathogenesis establishes a solid rationale for implementing strategies that are dedicated to restoring barrier function. Emollients are already essential in AD management [84], serving as a first-line therapy and having a potential steroid-sparing effect [85,86,87]. Given their potential to improve skin barrier integrity and possibly block the consequent exposure and inflammatory cascade of AD, skin barrier enhancement interventions have also undergone intensive research. After the results of some small, pilot, randomised, controlled trials suggested that the preventive application of emollients can be an effective strategy for hAD primary prevention [88,89,90], larger randomised and controlled trials on prevention have been coordinated to test if this strategy can prevent AD [58,59,91,92,93,94,95,96,97,98,99,100], some of which are still currently underway [59,95,99]. While some of these studies found no significant preventive efficacy [58,91,93,94,97], a recent systematic review and meta-analysis concluded that the continual prophylactic use of emollients in early infancy may prevent or delay hAD, especially in high-risk populations [101]. Another review has found that the continuous use of prophylactic emollients, starting within a critical window of the first six weeks of life, reduces the probability of early-onset hAD [102]. More recently, a randomised, blinded, parallel, three-group, phase II trial showed that group B (once-daily application of a specific emollient) infants tended to maintain intact skin for a longer period, and the risk of AD tended to be lower [100].

However, the heterogeneity of these studies does not allow the overall generalisation of any conclusions [102]. The wide variation in emollients used, ranging from simple oil- or lipid-based [58,93] to gels or creams [91], emulsions [89], and ceramide-containing emollients [92], may have contributed to the variation in results [103]. Chalmers et al. (2020) suggest that a more sophisticated and complex emollient formulation, including bioactive ingredients, might potentially have a protective effect [58]. It is also reasonable to assume that the different product formulations could have contributed to the controversial results [104]. Furthermore, considering the diversity of barrier dysfunction mechanisms, these conflicting results also indicate that, similarly to hAD treatment, there is no “one-size fits all” in its prevention, and further research on pathophysiology and biomarkers to develop more specifically tailored products is desired [102]. In conclusion, several uncertainties remain, namely which type of formulation should be used (simple versus complex), which ingredients should be included (or excluded) and their respective concentration, the optimal quantity for application (a standardised amount versus an individual approach), the recommended frequency of application (twice daily, daily, every other day, or others), the timing of application (pre- or post-bathing), the ideal age range to initiate the protocol, and the minimal duration of these protocols.

The novel knowledge of AD’s multifactorial aetiology offers a variety of points to pursue [48] for the potential development of more complex emollient products with bioactive ingredients, including anti-inflammatory agents and microbiome and immunological modulating effects [105], opening a new realm of possible therapeutic options. It is also important to note that a significant challenge of these studies was defining the application quantities and achieving parents’ compliance with the rigorous and lengthy protocol [58,91]. Therefore, further research should not only consider the emollient’s formulation but also optimise the protocol to maximise compliance.

### 3.3. Primary Prevention Strategies in Dogs

Conversely to our human counterpart, cAD primary prevention research is severely lacking. To the author’s knowledge, only two such studies have been performed on the subject, both falling in the category of interventions ingested by bitches and puppies [106,107,108].

Marsella (2009) proposed to evaluate the efficacy of the probiotic Lactobacillus strain GG (LGG) for alleviating or preventing cAD clinical signs [109]. The study used two adult Beagle dogs with severe disease and 16 of their puppies. These were kept in the same controlled environment (consistent temperature and humidity), with controlled allergen exposure and the same diet. Two litters were produced at a 1-year interval, the first forming the control group and the second the intervention group. The probiotic was administered to the bitch during the second pregnancy and to the second litter of puppies from 3 weeks to 6 months of age. Both litters were epicutaneously sensitised to a species of dust mite, Dermatophagoides farinae (DF), and the 6-month puppies underwent intradermal allergen testing and were challenged (exposed) to DF to evaluate clinical signs. In the control group, 7/7 puppies were strongly seropositive for IgE against DF, 6/7 had a positive reaction to intradermal testing, and 7/7 developed severe clinical signs. In the intervention group, 7/9 puppies were seropositive, 3/9 had a positive reaction to intradermal testing and 6/9 developed clinical signs. While LGG administration did not appear to decrease cAD clinical signs or prevent the disease’s development, it was associated with a reduced mean serum titre of IgE and reduced skin reactivity to intradermal injection of the allergen, therefore seeming capable of altering the cAD immune response.

Interestingly, a follow-up study was conducted three years after the discontinuation of LGG [107], where allergen-specific IgE, IL-10, and TFG-β were measured before allergen exposure and clinical signs were evaluated before and after allergen exposure. In this follow-up, the probiotic group developed lower clinical scores and had significantly less IL-10 for all allergens measured than the control group. The allergen-specific IgE and TGF-β did not differ between litters. It was concluded that early exposure to probiotics had long-term and sustained clinical and immunological beneficial effects in these dogs, and that additional studies with spontaneously atopic dogs are needed to better assess the protective effects of probiotics against cAD development.

In 2015, Beeck et al. investigated the effect of feeding pregnant Labrador Retriever bitches and their litters diets that were enriched in essential fatty acids, pantothenate, choline, nicotinamide, histidine, and inositol on owner-assessed cAD incidence [108]. Two owner questionnaires were used to assess the occurrence of cAD-associated clinical signs between the ages of 22 and 36 months and 36 and 48 months. The circulating IgE levels to two common dust mites, *Dermatophagoides farina* (DF) and *Dermatophagoides pteronyssinus* (DP), were measured when the puppies were 6 and 12 months old. During the first year of life, higher levels of circulating dust mite IgE were significantly higher in the control group than in the intervention group. Furthermore, at the time of the second questionnaire, the owner-assessed incidence of cAD was significantly lower in the intervention group when compared with the control group (2/24 (8.3%) dogs v. 10/33 (30.3%) dogs). The two positive dogs of the intervention group came from the same litter, while the ten positive dogs of the control group came from five different litters of the six enrolled. Furthermore, the rates of pruritus were significantly different between the groups, with more symptomatic animals in the control group. Although objective skin barrier parameters were not measured, the authors hypothesised that this benefit might be linked to improved barrier function and a consequent decreased percutaneous absorption of irritants, allergens, and microbes, preventing allergic sensitisation and inflammation.

Similarly to hAD, the reparation of the defective epidermal barrier is considered an important therapeutic goal in cAD’s management, and the use of lipid-containing products is recommended [7,14]. Furthermore, a previous study showed that an essential fatty acid supplementation had a steroid-sparing effect in atopic dogs, which could be explained by a possible skin barrier improvement [110]. Although extensive research exists on strategies directed at the external skin surface to improve skin barrier function in cAD management [1,14,28,39,67,111,112,113,114,115,116,117,118,119,120,121,122,123,124], there are, as yet, no studies on cAD prevention to the authors’ knowledge.

In 2011, Marsella et al. stated that “if future studies confirm that impaired skin barrier function in canine AD leads to increased allergen penetration and increased risk for allergic sensitisation, it is tempting to speculate that proactive restoration of the skin barrier could alter the course of the disease and minimise the development of an allergic response” [125]. Since then, Olivry et al. (2011) proved that SC removal facilitates experimental sensitisation to mite allergens in atopic dogs [126]. Therefore, skin barrier impairment has significant consequences on the cAD process as it increases the risk for sensitisation [126], leading to vicious cycles of sensitisation and additional inflammation, trauma, and skin damage [45]. A 2015 review [55] also concluded that there is considerable evidence associating abnormalities in SC lipids and proteins with impaired epidermal barrier function and clinical canine AD, and that future studies should focus on developing drugs that are able to restore the skin barrier. Another recent review [14] also asserts a great need for effective, easy-to-apply, and well-tolerated lipid-based topical products that are formulated explicitly for atopic dogs. Moreover, it claims that “the ability to prevent disease (cAD) in dogs through topical skin barrier repair has not been assessed but should be studied”. Therefore, cAD prevention through topical skin barrier repair is a needed, promising, and severely lacking research area in veterinary dermatology/medicine.

Additionally, cAD is a very heterogenous clinical syndrome [6,7,35], with ongoing research to characterise each subtype based on either epidermal barrier defects or endotypes [74,81,127,128,129,130]. Further characterisation of the various specific clinical phenotypes of cAD may optimise clinical outcomes by establishing what preventative and therapeutic strategies work best for each dog [7]. In doing so, veterinarians may start to move away from “one size fits all” or “trial by error” cAD management, and also potentially a prevention framework, to one defined by personalised, precision medicine [7].

## 4. Conclusions and Future Perspectives

In summary, cAD primary prevention is a much-needed, desirable, and promising research area. The development of effective and safe cAD primary prevention strategies would be of invaluable interest and the utmost importance, primarily for the QoL of dogs and their caregivers. It could represent a turning point in how many canine patients and their families unavoidably suffer a life-long and very distressing disease. Furthermore, considering that cAD is an excellent model for hAD [1,23,25,26,106,131,132] and given the shorter lifespan of dogs when compared to humans, and especially the time lapse from birth to adulthood (1–2 years versus 18 years, respectively), this type of study can also contribute to a faster production of evidence-based scientific output, applicable in human research, especially in paediatrics. Also, in addition to improving health and well-being, it could also benefit economic and sustainability resources, from the research field to the diagnostic and treatment areas.

Any research on this exciting area would be welcome and valuable, especially in a clinical, pragmatic context, for although the comprehension of the mechanisms through which the effects of prevention are mediated is interesting, it is not, at this point, essential [37].

## Data Availability

No new data were created or analysed in this study. Data sharing is not applicable to this article.
